# Reducing Microvascular Dysfunction in Revascularized Patients with ST-Elevation Myocardial Infarction by Off-Target Properties of Ticagrelor versus Prasugrel. Rationale and Design of the REDUCE-MVI Study

**DOI:** 10.1007/s12265-016-9691-3

**Published:** 2016-04-21

**Authors:** Gladys N. Janssens, Maarten A. H. van Leeuwen, Nina W. van der Hoeven, Guus A. de Waard, Robin Nijveldt, Roberto Diletti, Felix Zijlstra, Clemens von Birgelen, Javier Escaned, Marco Valgimigli, Niels van Royen

**Affiliations:** Department of Cardiology, Institute of Cardiovascular Research ICaR-VU, VU University Medical Center, De Boelelaan 1117, 1081 HV Amsterdam, The Netherlands; Department of Cardiology, Erasmus Medical Center, Rotterdam, The Netherlands; Department of Cardiology, Medisch Spectrum Twente, Thoraxcentrum Twente and Health Technology and Services Research, MIRA Institute, University of Twente Enschede, Enschede, The Netherlands; Cardiovascular Institute, Hospital Clínico San Carlos, Madrid, Spain; Department of Cardiology, Swiss Cardiovascular Center Bern, Bern, Switzerland

**Keywords:** Ticagrelor, Prasugrel, Microvascular injury, ST-elevation myocardial infarction, Adenosine

## Abstract

Microvascular injury is present in a large proportion of patients with ST-elevation myocardial infarction (STEMI) despite successful revascularization. Ticagrelor potentially mitigates this process by exerting additional adenosine-mediated effects. This study aims to determine whether ticagrelor is associated with a better microvascular function compared to prasugrel as maintenance therapy after STEMI. A total of 110 patients presenting with STEMI and additional intermediate stenosis in another coronary artery will be studied after successful percutaneous coronary intervention (PCI) of the infarct-related artery. Patients will be randomized to treatment with ticagrelor or prasugrel for 1 year. FFR-guided PCI of the non-infarct-related artery will be performed at 1 month. Microvascular function will be assessed by measurement of the index of microcirculatory resistance (IMR) in the infarct-related artery and non-infarct-related artery, immediately after primary PCI and after 1 month. The REDUCE-MVI study will establish whether ticagrelor as a maintenance therapy may improve microvascular function in patients after revascularized STEMI.

## Introduction

Percutaneous coronary intervention (PCI) in patients with ST-elevation myocardial infarction (STEMI) leads to an improved patency rate of the infarct-related artery, a lower incidence of heart failure, and ultimately an improved survival as compared to thrombolytic treatment [1]. However, problems that frequently occur after restoration of the epicardial blood flow are inadequate myocardial reperfusion and microvascular injury (MVI) [2].

MVI can be visualized by non-invasive methods using cardiovascular magnetic resonance imaging (CMR) or measured by invasive methods such as intracoronary thermodilution [3]. MVI as assessed by CMR is related to left ventricular remodeling and clinical outcome after STEMI [4, 5].

From the acute to late phase of a revascularized myocardial infarction, several mediators are thought to contribute to the development of MVI. These factors involve the appearance of intraluminal platelets, fibrin thrombi, and neutrophilic granulocytes, resulting in inflammatory cell plugging, formation of microthrombi, endothelial activation and injury, and finally MVI. Vasoconstriction follows as a result of endothelial dysfunction with decreased levels of nitric oxide and increased levels of endothelin-1. Furthermore, the imbalance in coagulation factors and influx of inflammatory cells can cause vascular leakage and hemorrhage [6, 7]. Adenosine, a substance naturally present in the blood, has an inhibitory effect on several of these processes [8, 9].

Currently, in patients with STEMI, the P2Y_12_-receptor antagonists ticagrelor and prasugrel, in addition to aspirin, are the recommended antiplatelet drugs according to European and American guidelines [10, 11]. The efficacy of ticagrelor and prasugrel in reducing platelet activity is comparable [12]. The long-term effects on clinical outcome are presently subject to investigation [13]. Interestingly, ticagrelor, besides its antiplatelet effects, increases adenosine concentration in the blood plasma by inhibition of intracellular reuptake and might therefore have an additional beneficial effect by preventing MVI [14–17]. Therefore, we designed a randomized study, comparing the effects of ticagrelor and prasugrel on MVI and endothelial dysfunction after STEMI.

## Methods

**Fig. 1:**
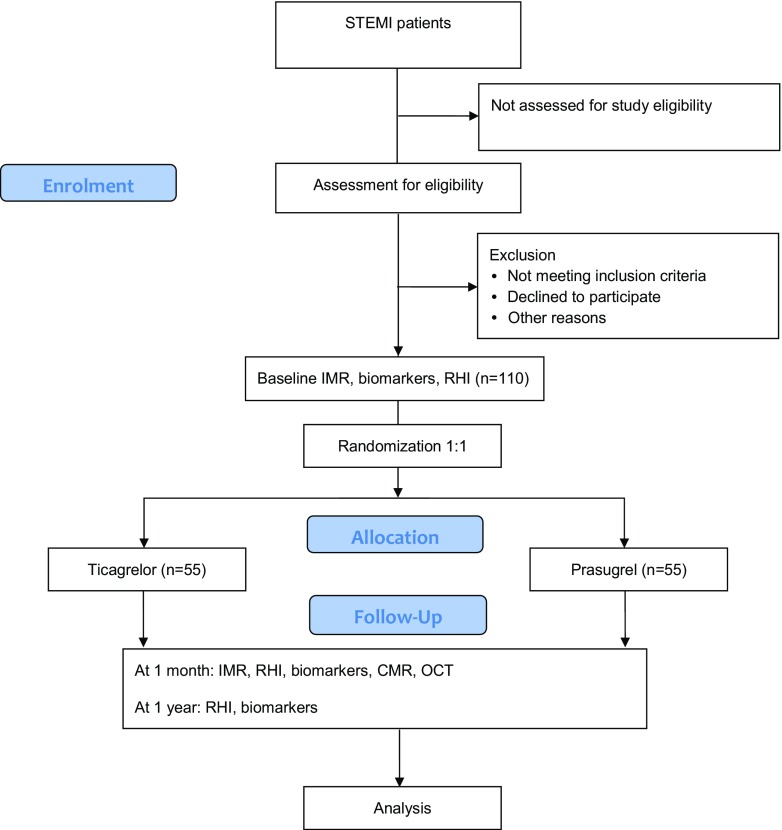
Flow chart of the REDUCE-MVI study. *CMR* cardiovascular magnetic resonance, *IMR* index of microcirculatory resistance, *OCT* optical coherence tomography, *PCI* percutaneous coronary intervention, *RHI* reactive hyperemia index, *STEMI* ST-elevation myocardial infarction

The Reducing Micro Vascular dysfunction In revascularized ST-elevation myocardial infarction patients by off-target properties of ticagrelor (REDUCE-MVI) study is a multicenter trial with a prospective, randomized, open-label, blinded-endpoint (PROBE) study design. Current participating centers are the VU University Medical Center (Amsterdam, the Netherlands), the Erasmus Medical Center (Rotterdam, the Netherlands), the Medisch Spectrum Twente (Enschede, the Netherlands), and the Hospital Clínico San Carlos (Madrid, Spain). A flow chart of the study design is shown in Fig. 1.

## Patient Enrolment

On hospital arrival, patients presenting with an acute STEMI will be screened for participation in the study according to the inclusion and exclusion criteria (Table 1). ST-elevation criteria are used according to current ESC guidelines [10]. After successful PCI, witnessed oral informed consent will be obtained and documented, before proceeding with study measurements. After the procedure, written informed consent in native language will be obtained from all individual participants that will be included in the study. Hereafter, patients will be randomized to treatment with either ticagrelor or prasugrel. A total of 110 patients will be enrolled in the study.

**Table 1:** Inclusion and exclusion criteria

Inclusion criteria	Exclusion criteria
Acute STEMI <12 h The patient has received a loading dose ticagrelor 180 mg before start of PCI Successful PCI of the infarct-related artery with a modern DES Intermediate stenosis in a non-infarct-related artery (50–90 %) Provision of informed consent	Age <18 or ≥75 Body weight <60 kg History of myocardial infarction, coronary artery bypass graft, stroke, or transient ischemic attack Congestive heart failure, left ventricle ejection fraction <35 % Severe liver or kidney dysfunction Bleeding diathesis, platelet count <100,000/mm^3^ Indication or use of (novel) oral anticoagulant therapy Cardiogenic shock Chronic total occlusion, left main disease Inability or contra-indication for MRI Inability to be followed on-site Limited life expectancy

*DES* drug-eluting stent, *MRI* magnetic resonance imaging, *PCI* percutaneous coronary intervention, *STEMI* ST-elevation myocardial infarction

## Coronary Angiography and Revascularization at Index and 1 Month

PCI will be performed according to standard procedures and is left to the discretion of the operator. All patients will receive a loading dose of heparin, aspirin, and the P2Y_12_ inhibitor ticagrelor in the ambulance before primary PCI with a third-generation drug-eluting stent (DES) (standard of care in participating centers). After successful primary PCI, myocardial tissue perfusion of the infarct-related area will be assessed by determining the myocardial blush grade [18].

After 1 month, patients will undergo clinically indicated FFR-guided PCI of the intermediate lesion in the non-infarct-related artery.

## Pharmacologic Treatment

Patients will be randomly assigned to use either ticagrelor 90 mg BID or prasugrel 10 mg SID as a maintenance therapy continued for 1 year according to current international guidelines [19]. Concomitant medical therapy will be left to the discretion of the treating physician.

## Endpoints

The primary objective of this study is to determine whether ticagrelor at treatment steady-state in revascularized STEMI patients is associated with an improved microvascular function as measured with IMR, compared to prasugrel. Both treatment groups are subjected to the same measurements at three time points. The function of the coronary microcirculation is considered as primary endpoint which will be assessed by determining the index of the microcirculatory resistance (IMR) [3]. Microcirculatory resistance measurements of the infarct-related artery and secondarily of the non-infarct-related artery will be performed directly after primary PCI and after 1 month. Secondary endpoints will be coronary endothelialization, left ventricular function, and infarct size at 1 month, as well as peripheral endothelial function and several biochemical markers of endothelial function, measured at baseline and at follow-up after 1 month and 1 year.

## Index of Microcirculatory Resistance

**Fig. 2:**
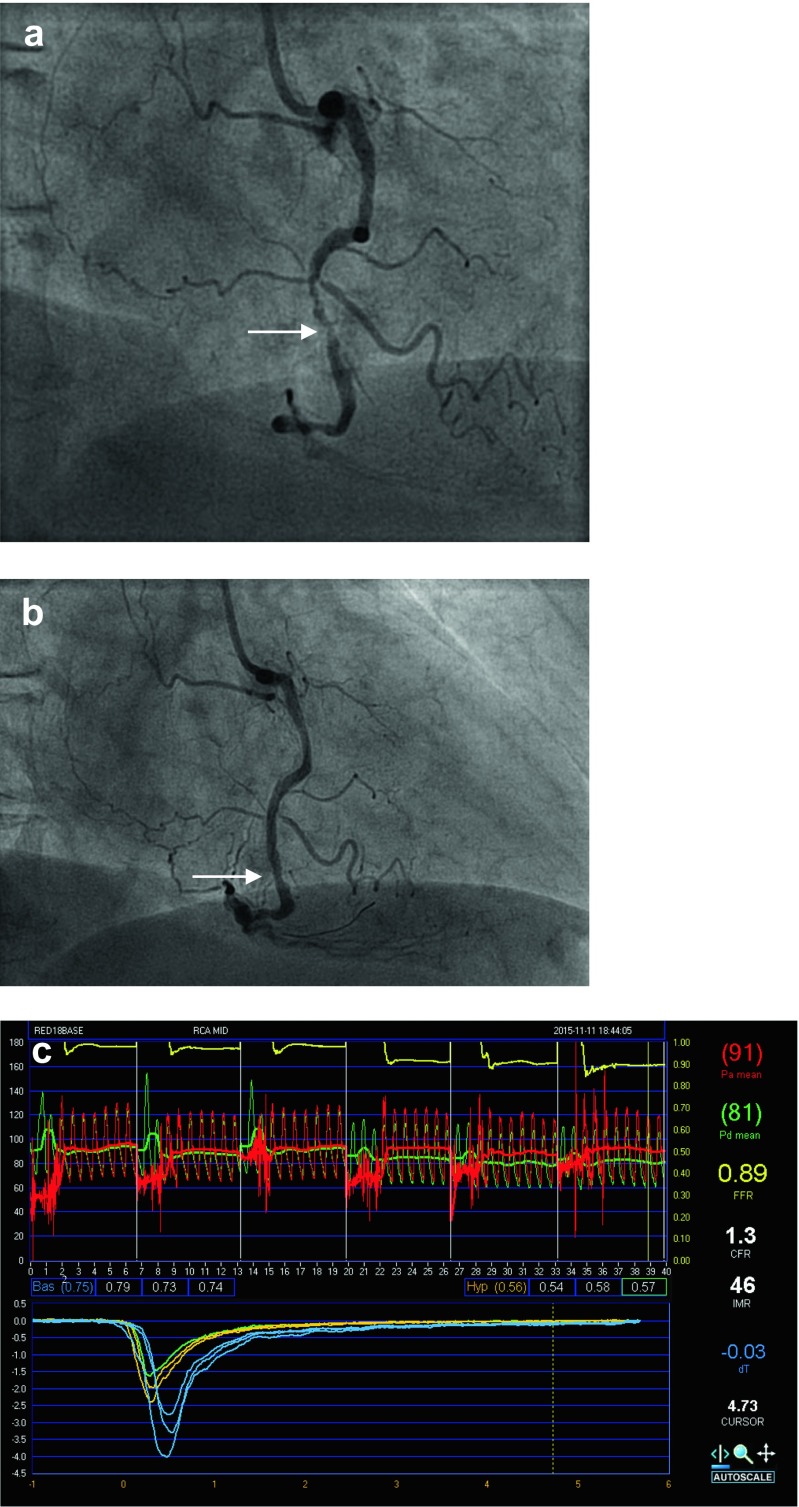
Primary PCI procedure with subsequentmeasurement of the IMR. **a** Angiographic demonstration of the presence of a subtotal occlusion of the right coronary artery (RCA) in its mid portion and **b** final result after successful reopening of the RCA with subsequent implantation of two DES stents; **c** measurement of the IMR. The recording is obtained from a pressure wire in the mid of the right coronary artery of a patient after primary PCI. The *panel* is divided into two *windows*; the *upper window* displays the pressure segments recorded during each saline injection, separated from each other by *white vertical lines* (mean proximal arterial pressure (P_a_) = 91 and mean distal arterial pressure (P_d_) = 81 result into a fractional flow reserve (FFR) = 0.89). The lower graph displays saline injections at baseline (*blue*) and during hyperemia (*yellow*). Between the two graphs are mean transit time values at baseline (preceded by “Bas”), and during hyperemia (preceded by “Hyp”)

IMR in the infarct-related artery will be measured after 1 month and compared to IMR immediately after primary PCI. Secondly, IMR in the non-infarct-related artery will be determined after 1 month and compared to measurements at baseline (Fig. 2).

By using a coronary pressure and temperature-sensitive guidewire (Certus™, ST Jude Medical, Uppsala, Sweden) and modified software, the transit time of room-temperature saline injected in a coronary artery can be determined. After an intracoronary injection of 200 mcg nitroglycerine to establish epicardial coronary vasodilatation, the guidewire is calibrated outside the body, equalized with aortic pressure at the ostium of the guide catheter, and then advanced into the distal third of the artery and distal to the stent in the infarct-related artery. The mean transit time at baseline is obtained by administering three times 3 mL of intracoronary saline. Thereafter, hyperemia is induced by administering adenosine at 140 mcg/kg per minute intravenously and measurement of mean transit time is repeated. Aortic and distal coronary pressures are recorded simultaneously. The distal coronary pressure divided by the inverse of the hyperemic mean transit time then provides the IMR. This calculation will be performed by independent and blinded analysts in a core laboratory.

## Optical Coherence Tomography

Optical coherence tomography (OCT) will be performed at 1 month with a Dragonfly™ OCT catheter (St Jude Medical, St. Paul, MN, USA) using a motorized pullback device. OCT pullbacks will be performed at a speed of 20 mm/s in the infarct-related and non-infarct-related arteries, using in general a contrast flush rate of 3 mL/s in the right coronary artery and 4 mL/s in the left coronary artery. The occurrence of subclinical stent thrombus, stent malposition, and edge dissections will be assessed. All acquired images are sent to a core laboratory for blinded evaluation (endothelial aspects of the infarct- and non-infarct-related artery) using a standardized protocol.

## Peripheral Endothelial Function

The peripheral endothelial function will be measured within 24 h after primary PCI, at 1 month, and 1 year follow-up.

EndoPAT^®^ (Itamar Medical Ltd., Caesarea, Israel) is a validated device indicated for non-invasive endothelial and microvascular function assessment [20–22]. EndoPAT^®^ will be used to measure the finger arterial pulsatile volume changes that indicate changes in vascular tone, by modified plethysmographic probes placed on the index finger of each hand [23]. The measurements will be performed at rest in a convenient, low stimulus environment. Each recording consists of subsequently 5 min of baseline measurement, 5 min of occlusion time of the brachial artery by a blood pressure cuff, and 5 min post-occlusion measurement (hyperemic period). Measurements will be performed on the contralateral arm of the access site of angiography. The access site extremity is used as a reference to control for concurrent non-endothelial-dependent systemic changes in vascular tone.

The reactive hyperemia index (RHI) and its natural logarithm are then calculated as the ratio of the magnitude of the average post-occlusive pulse volume amplitude to the average pre-occlusive pulse volume amplitude, corrected for baseline vascular tone.

## Cardiovascular Magnetic Resonance

After 1 month and optionally at baseline (day 2–7), CMR will be performed with a 1.5 or 3.0 Tesla scanner using a phased array cardiac receiver coil. All images are ECG-gated and acquired during mild end-expiration breath-holding. Cine long- and short-axis slices are obtained to examine regional and global left ventricular function, volumes, and ejection fraction. Evaluation of myocardial ischemia will be assessed with first-pass perfusion imaging of a gadolinium-based contrast agent during the administration of 140 mcg/kg per minute of adenosine intravenously. Finally, late gadolinium-enhanced (LGE) images are acquired 10 min after perfusion imaging, to identify the size and extent of myocardial infarction and, in addition, the presence and extent of MVI. When performed at baseline, T2-weighted imaging will be acquired in short-axis planes for the detection of intramyocardial hemorrhage and infarct-related edema. All CMR analyses will be performed in a core laboratory by blinded observers using a standardized protocol.

## Blood Collection and Storage

Blood samples for laboratory testing will be drawn at baseline and at 1 month and 1 year follow-up according to standard procedures. The testing includes standard biochemistry and hematology tests (e.g., renal function tests, cardiac biomarkers, lipid profile, and blood cell counts). Furthermore, extra blood samples will be collected and stored at −80 °C. At the end of the study, all samples will be analyzed by the laboratory at the VU University Medical Center. The plasma levels of ticagrelor will be examined as well, and P2Y_12_ inhibition levels will be assessed by VerifyNow testing (Accumetrics, San Diego, CA, USA). The effect of ticagrelor on inflammatory markers and cells, such as cell adhesion molecules and monocytes, will be studied concomitantly. In addition, the level of biochemical markers for endothelial function, like asymmetrical dimethylarginine, will be measured, which allows linking these results to the microvascular function.

## Registration of Adverse Events

Adverse events (AEs) are evaluated and registered during index hospitalization and at 1 month and 1 year follow-up. AEs occurring in external participating centers will be reported to the sponsor. A serious adverse event (SAE) is a severe undesirable event in which there was no necessarily causality with the study. The relationship of SAEs to study treatment or procedures will be assessed by the investigators and communicated to AstraZeneca.

As a safety measure, bleeding complications will be monitored during the entire study period, categorized to the most recent criteria (Bleeding Academic Research Consortium, Thrombolysis in Myocardial Infarction [24], and Global Use of Strategies to Open Occluded Coronary Arteries [25]) and analyzed for both treatment groups.

Once a year, the sponsor will submit a safety report to the accredited medical ethics committee and competent authority. After 50 % inclusion, an interim analysis will be performed, with respect to safety endpoints, AEs, and SAEs. An independent data safety monitoring board, composed of experienced cardiologists and a statistician, will be responsible for reviewing patient safety and study integrity.

## Sample Size Calculation

The study is powered using a superiority design with the null hypothesis that IMR will be significantly improved in the ticagrelor group in comparison to the prasugrel group. A difference of IMR means of 7 at 1 month follow-up between the two treatment groups, with a standard deviation of 12, is expected [26]. To be able to reject the null hypothesis that the means of both treatment groups are equal with a power of 80 % (with significance level *α* = 0.05), 47 subjects in each group are required. Assuming a loss of follow-up of maximally 15 %, it will be necessary to include a total of 110 patients in the study. After 50 % inclusion (*n* = 55) with complete 30-day follow-up, an interim analysis will be performed to verify the assumed standard deviation and, if necessary, we might adjust the total sample size needed to achieve an adequate power.

## Discussion

Optimal therapy of STEMI requires both treatment of the macrovasculature and the microvasculature. Over the years, treatment of the macrovasculature has improved notably. Due to refined stent designs and more biocompatible polymers on DESs, the prevalence of acute stent thrombosis and restenosis has been reduced significantly. However, inadequate myocardial perfusion after restoration of the epicardial blood flow by primary PCI is present in a large proportion of STEMI patients. Nearly 50 % of the final myocardial infarct size is due to myocardial reperfusion injury, and thus, a logical target for reducing myocardial infarct size, thereby maximizing the benefits of reperfusion [27].

The cornerstone of treatment of patients after STEMI is antithrombotic therapy with a P2Y_12_-receptor antagonist and aspirin. According to current ESC and ACCF/AHA guidelines, both ticagrelor and prasugrel have a class 1B recommendation for use after STEMI [10, 11]. Both of these drugs have advantages over clopidogrel as they achieve a more rapid, consistent, and stronger platelet inhibition than clopidogrel with improved outcomes [28, 29]. With regard to the efficacy in inhibiting platelet function, ticagrelor and prasugrel have comparable effects [12]. Currently, the ISAR-REACT 5 study is enrolling patients to compare ticagrelor and prasugrel for clinical outcome [13].

It has been shown that ticagrelor, besides its antiplatelet effects, also has the potency of increasing the plasma concentration of endogenous adenosine in patients with acute coronary syndromes. Adenosine is produced particularly after tissue damage by hypoxia, and ticagrelor has the ability to inhibit intracellular uptake through the adenosine equilibrative nucleoside transporter 1 (ENT1). Prasugrel and clopidogrel, two other P2Y_12_-receptor antagonists, do not mediate such conservation of the plasma adenosine concentration [15, 16]. This indicates that ticagrelor might be a superior drug for treating STEMI.

There is evidence that elevations of adenosine reduce the inflammatory response in the different phases after revascularized myocardial infarction, by interfering with the production of inflammatory mediators and oxygen species and neutrophil trafficking [8, 9].

In the acute phase of myocardial infarction, myocardial damage is enhanced by the formation of microthrombi as a result of a hypercoagulable state and by inflammatory cell clogging of the intramyocardial microvasculature. In the early phase (hours to days), infiltrating cells further amplify damage, as an influx of blood-borne inflammatory cells leads to production of inflammatory mediators and tissue-destructive agents such as metalloproteinases [30]. In the late phase of myocardial infarction (days to months), which is characterized by irreversible damage of the microcirculation, adenosine might also have beneficial effects, as it stimulates at elevated levels angiogenesis by the induction of endothelial progenitor cell migration [31]. Only effective angiogenesis and arteriogenesis will be able to restore microvascular and macrovascular tissue perfusion to prevent increased scarring. Furthermore, adenosine may have a stimulatory effect on endothelial healing, which may be particularly favorable in patients who underwent successful primary PCI for STEMI.

In these ways, adenosine might reduce ischemia and reperfusion injury, preserving microvascular function. This might be most important for patients with STEMI, as coronary MVI and dysfunction appear to be most pronounced in this patient group, compared to patients with non-ST-elevation acute coronary syndrome [32].

In the REDUCE-MVI study, we aim to investigate the potential adenosine-mediated effects of ticagrelor with several outcome parameters, especially IMR, to determine the effect on coronary microvascular function.

In the coming years, more insight into the biochemical mechanisms involved in STEMI may be expected, which may hopefully lead to the development of more specific drugs for microvascular preservation. Along this line, the REDUCE-MVI study is focused in more detail on current therapeutic options to retain microvascular integrity and function in patients with STEMI.

## Abbreviations

AE: Adverse event
CMR: Cardiovascular magnetic resonance
DES: Drug-eluting stent
FFR: Fractional flow reserve
IMR: Index of microcirculatory resistance
MRI: Magnetic resonance imaging
MVI: Microvascular injury
OCT: Optical coherence tomography
PCI: Percutaneous coronary intervention
RCA: Right coronary artery
RHI: Reactive hyperemia index
SAE: Serious adverse event
STEMI: ST-elevation myocardial infarction
